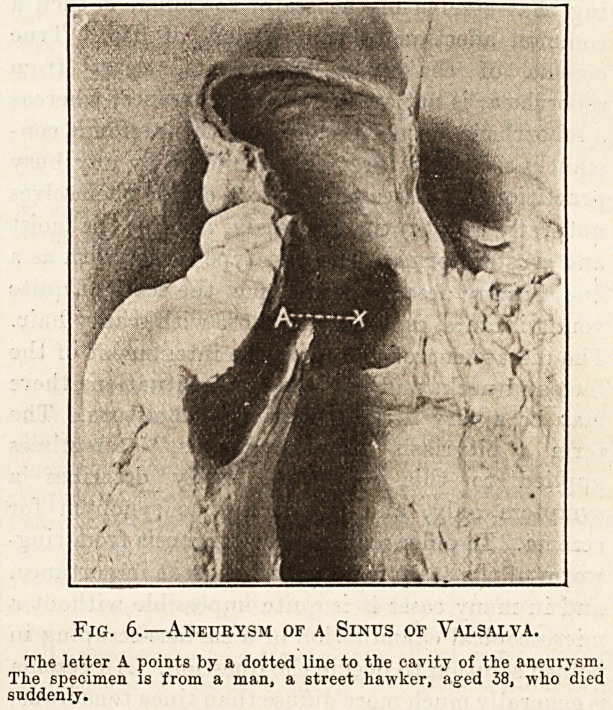# Some Affections of the Heart Concerned in Sudden Death

**Published:** 1907-05-18

**Authors:** Theodore Fisher


### Hospital Clinics.

### SOME AFFECTIONS OF THE HEART CONCERNED IN SUDDEN DEATH.

By THEODORE FISHER, M.D., M.R.C.P.

### IV. THE CARDIAC MUSCLE—(*continued*).

There are other causes of fibroid disease of the heart besides disease of the first part of the aorta and atheroma of the coronary arteries. Syphilis may give rise to this affection of the heart both through the medium of disease of the arteries and gummata. It has never fallen to my lot to meet with a case where fibrosis of the heart appeared to have followed disease of the coronary arteries of clearly syphilitic nature, but large fibrous patches which with little doubt have been the sequel of the presence of a gumma are not very uncommon. I have seen a very large patch of this character in the heart of a prostitute, who, however, did not die suddenly. Yet not only these large patches, but gummata in their earlier stages may occasion sudden death.

### A Case of Syphilitic Disease.

It may be interesting to refer to a case of syphilitic disease of the heart which came under my notice where death, although not absolutely sudden, was of acute onset. On calling one afternoon at a hospital in order to see one of the residents, I was taken to see a case which had recently been admitted. The patient, a finely-built captain of a ship, was said to have had several fits, and one of these fits occurred soon after we entered the ward. He became intensely cyanosed, no pulse could be felt, and no cardiac sounds were audible. There was a deep respiration about every half-minute, sometimes, perhaps, at a shorter, sometimes at a longer interval. The pupils were somewhat contracted. After four or five minutes had elapsed, during which time it looked as if every moment were to be his last, he suddenly became flushed and the heart could be felt beating forcibly. The pulse was then about 80 to the minute and for a time regular. The impulse, however, soon grew feebler and the pulse irregular, the intermittence, however, varying with the respiration, occurred for a time at regular intervals, every fourth beat being absent. After another few minutes had passed the pulse grew regular, but was slow—only 44 to 46 to the minute. During this time the respirations had become natural, but the patient was restless and worked himself into the sitting posture. A little later, about twenty minutes after the onset of the fit, he was semi-conscious. On examination of the heart a loud widely-conducted pulmonary systolic murmur was now audible, and also at the apex the dull diastolic sound, which sometimes has not inaptly been called "the third sound" of the heart. Several similar fits followed during the course of the evening and the man was dead before the morning. At the post-mortem examination a gumma was found in the septum interventriculorum immediately below the aortic valve. It was the size of a large filbert-nut and projected into the cavities of both ventricles. For many years it has been noticed that lesions of the septum interventriculorum are more likely to occasion serious disturbance of cardiac action and sudden death than areas of disease elsewhere in the cardiac muscle. Apparently, however, only comparatively recently has the possibility of interference with the bundle of His, which passes from the right auricle to the septum between the ventricles in this situation, been thought to be the explanation of these disturbances. The above case is a good illustration of the sensitive character of the upper portion of the septum interventriculorum, and also of the combination of symptoms which are known by the name of Stokes-Adams' disease.

A lesion of the heart-wall was present here, which led to death, not suddenly, but in the course of a few hours. Death, however, may be absolutely sudden. A doctor whom I once met said he was talking to his father, who was preparing to start on his morning's professional round, when he fell dead, and that to all appearance he was dead before he reached the ground. In other cases there may be a short ejaculation indicating a sense of apprehension of evil, or death may be preceded by a sense of faintness. Again, a condition of collapse may exist somewhat resembling the result of an abdominal injury, or there may be distressing attacks of severe cardiac pain. Whether death be absolutely sudden or delayed for a few minutes, or even hours, some variety of fibroid disease of the cardiac muscle will, in the majority of instances, be found after death. There are, however, exceptions to this rule. Occasionally, especially in very old people, the heart will present no obvious lesions. In such cases cessation of cardiac action is probably due to the age of the heart-muscle. It has been unable to withstand some slight strain, or possibly disturbance by some toxin, which at an earlier period of life would have been harmless. But even in middle life it may occasionally happen that the heart may fail, yet nothing abnormal can be detected. On one occasion I was asked to perform a post-mortem examination on a man, aged about 35, who had returned home, saying that he did not feel well, sat down in a chair, and died. Nothing whatever could be found in the heart or elsewhere to account for death. Possibly such cases of cardiac failure are toxic in nature.

Stress has been laid upon the fact that fibroid disease of the heart is the most common cause of sudden death. This is well recognised, but it is perhaps not so well known that in cases of sudden death nothing but a large heart may be found in which disease of the cardiac muscle and of the valves is absent. It is these cases which are most likely to be considered instances of death from mitral regurgitation. To those who are unaware that large hearts of this character may fail suddenly it is easy to commit the error—referred to in a previous article—of mistaking normal thickening of the edge of the mitral valve for disease, especially as some text-books speak of mitral regurgitation as a cause of enlargement of the heart.

Occasionally the large heart may be found associated with a red granular kidney, but more commonly the kidneys are healthy. Possibly the most common cause of a large heart, where valvular disease and chronic interstitial nephritis are absent, is alcoholic intemperance. Cases of sudden death are by no means uncommon, where circumstances suggest that this is the most probable cause for the enlargement. For example, a carter, aged between 30 and 40, falls dead off the seat of his wagon while driving. A large heart is found, weighing 16 ounces, but lesions of the valves, disease of the cardiac muscle, and of the kidneys are absent. In such a case as this the occupation of the deceased man suggests that over-indulgence in alcohol was the probable cause of the enlargement. In other cases, when

Fig. 4.—The Large Heart of a Gas Stoker.

The smaller heart is of normal size, and is placed by the side of the larger for comparison. The age of the gas-stoker was 44.

death is not absolutely sudden, the condition of the dying man may possibly incorrectly lead the police to think that he is intoxicated. As an illustration a case of a man, aged 30, may be mentioned who was found "drunk" by the police. He died about half an hour later, and at the autopsy examination of his heart showed it to be much enlarged and weighing 18 ounces; but no cause could be found for the enlargement. Here alcohol may have been the cause of the enlargement, but recent heavy drinking was not the reason of rapid failure. In such a case, although the supposed drunken man proved to be dying of cardiac failure, it may be interesting to mention that a weak heart may fail while a man is taking alcohol. For example, a man, aged 64, became collapsed while drinking in a public-house, and died very shortly after. His heart proved to weigh as much as 20 ounces, but in this instance red granular kidneys were present, and the cardiac enlargement was probably not consequent upon alcoholic intemperance.

### Hard Work as a Factor in Enlarged Heart.

To return to the cases of enlarged heart in which renal disease is absent and there is no valvular lesion or fibroid degeneration of the cardiac muscle, although in cases of this nature where sudden death occurs, there are rarely means of ascertaining the part played by alcohol in producing the enlargement of the heart, alcohol is without doubt the most common cause of such enlargement, and in any individual case what cannot be proved may be considered highly probable. There are, however, other causes for the enlargement, one of which is arduous work. Some instances which have come under my notice seem to indicate that it is not laborious exertion merely, but such exertion in a hot atmosphere that is likely to cause the development of these large hearts. At least the best-marked examples I have met with have occurred in two instances in gas stokers, and in a third in a black-

Fig. 5.—Ball-thrombus in the Appendix of the Righ[illegible] Auricle.

smith. Fig. 4 is a photograph of the large heart of a gas stoker, and a heart of normal size placed by its side for comparison. The heart of the gas stoker weighed 35½ ounces. In none of these three cases, however, was death very sudden, but in one death took place in about 36 hours from the onset of the symptoms, extreme cyanosis being a marked feature. It may be interesting to mention in this connection that the report of an inquest in a daily paper once attracted my attention, which mentioned that a striker in engineering works, aged 34, had fallen dead while playing cricket, having just hit the ball for five runs. It occurred to me [although, since no post-mortem examination was made, it is needless to say there was no proof of the truth of the idea] that this man might have possessed a heart enlarged as the result of his hard work in the hot atmosphere of the factory.

A rare but remarkable cause of death is rupture of the heart. This cause of death seems to be especially common in insane patients. Mr. Cecil Beadles has recorded several such cases, which occurred in Colney Hatch Asylum. The cause of the rupture in some instances does not seem to be clear, though in others it appears to be due to local degeneration of the cardiac muscle dependent upon interference with the circulation. Another uncommon cause of sudden death is thrombosis of a coronary artery. Curiously enough, as Dr. Parkes Weber points out in a case which came under his notice, and I believe the same observation had previously been made by Dr. Moxon, there may be evidence in the thrombus that life has continued sufficiently long after the thrombus has formed to allow the clot to become partially organised. I have, however, met with a case where both coronary arteries were thrombosed. It is needless to say that death must have followed the thrombosis quickly in this instance. Still another cause of sudden death may be mentioned, of which Dr. Osler and others have recorded instances; this is, the detachment of a ball-thrombus from the appendix of one of the auricles and its fixation in the corresponding mitral or tricuspid orifice. Although no such accident happened in the case from which fig. 5 is taken, a glance at the illustration will make it clear how easily the circulation could be stopped by the presence of the ball-like clot in the tricuspid orifice which lies below.

### Aneurysm.

One other cause of sudden death may be mentioned, because, although the disease which occasions it is not within the heart, it lies within the boundaries of the pericardium. This cause of death is aneurysm of a sinus of Valsalva. Such an aneurysm produces no symptoms until it bursts, and when it bursts into the pericardium it leads to very rapid death. Occasionally an aneurysm in this situation may burst into the superior vena cava or pulmonary artery, and when this takes place death may not occur for some weeks, or perhaps a longer period, after the accident. In such cases interesting murmurs may be heard during life, but these do not concern us here. As an example of rapid death, the following case may be mentioned. A man, aged 46, awoke at 4 A.M. complaining of pain in the chest. He became collapsed and died at 7.30. At the autopsy an aneurysm the size of a walnut was found in one of the sinuses of Valsalva which had burst in the pericardium, where 15 ounces of blood were present. An illustration has been given of one of these small aneurysms (see fig. 6). In this case, curiously enough, although absolutely sudden death occurred, bursting of the aneurysm was not the cause. The possible mistakes of others have been mentioned, and I must confess my own. The specimen was removed from one of the first cases of sudden death upon which I made a post-mortem examination. Although aware of the importance of fibroid disease of the heart, I did not then realise the frequency with which disease of the aorta, by causing interference with the circulation

Fig. 6.—Aneurysm of a Sinus of Valsalva.

The letter A points by a dotted line to the cavity of the aneurysm. The specimen is from a man, a street hawker, aged 38, who died suddenly.

through the coronary arteries, leads to degeneration of the cardiac muscle. The specimen was mounted without extensive cutting into the cardiac muscle. The heart has remained untouched since, but there can be no doubt that the cause of death is to be found in the muscle-wall. While, however, this aneurysm did not rupture, it is a good illustration of yielding of the aorta over a small area immediately above the valve, and from its size it must be clearly realised how such a serious condition may exist without giving rise to symptoms, and the first indication of its existence be rupture ending in rapid death.

## Figures and Tables

**Fig. 4. f1:**
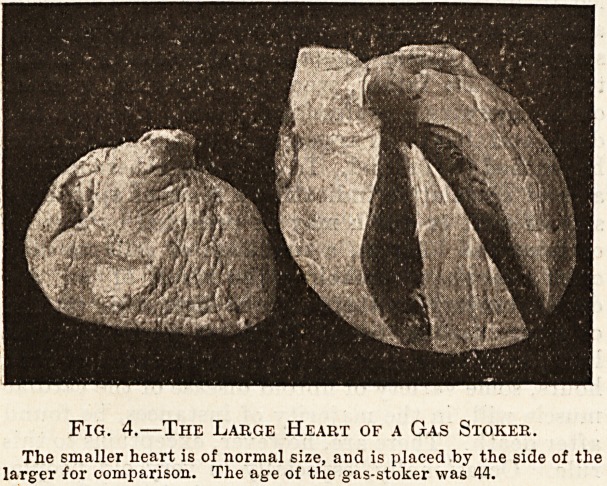


**Fig. 5. f2:**
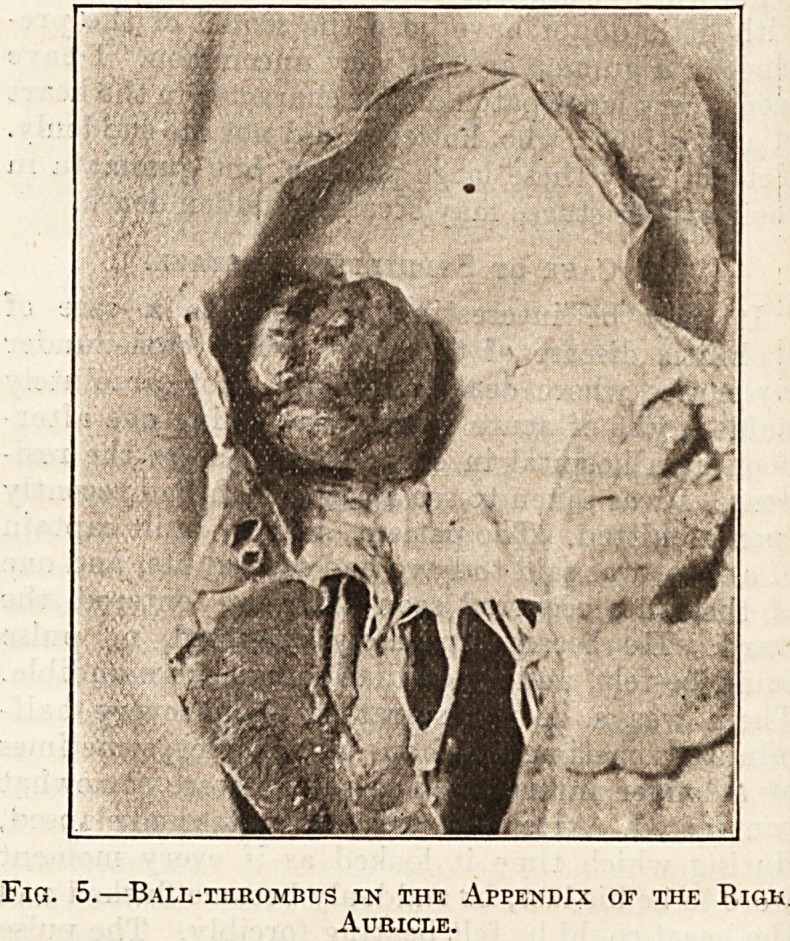


**Fig. 6. f3:**